# Structure of the Uterus

**Published:** 1843-08

**Authors:** 


*Structure of the Uterus.*—Jobert conceives that the peritoneum is united to the surface of the uterus by true muscular fibres; and that the uterus consists of a single muscle, whose fibres, arranged in super-imposed layers, run in the following directions:

1. The superficial longitudinal fibres, which may be called *median*, as they occupy the central raphé of the body of the uterus, rarely exist on its anterior surface; they are constantly found on its posterior aspect, where they consist of two thin super-imposed layers, commencing at the fundus of the uterus, and running to the uterine extremity of the vagina, to which they are attached, with the exception of a few which terminate on the neck of the uterus above the opening of the vagina. They adhere on the one hand to the peritoneum, on the other to the oblique fibres.

The superficial fibres of the anterior wall of the uterus form a layer covered by the peritoneum and lying on the deep fibres; they are so disposed that they do not embrace the entire surface of the wall of the uterus which they concur in forming, but they cross, before they reach the round ligament of the opposite side. Some of its fibres enter into the composition of the round ligament, while others pass behind it and terminate on the sides of the organ where they decussate with those from the posterior surface.

3. The remaining superficial fibres appertain to the tubes and to the ligaments of the ovaries: they are only apparent during pregnancy. Some arise from the fundus of the uterus, adhere to those which belong to the tubes, and run to the anterior part of the ligament of the ovaries, being slightly twisted on themselves; others more numerous, at first divergent, arise from the posterior surface of the fundus of the uterus, and also run to the ligament of the ovary. Finally, some transverse fibres, arising from the posterior surface, constitute the inferior portion of the organ.

The neck of the uterus is composed of the same tissue as the body. The fibres composing it represent semicircles, and decussate without intermixing in the direction of the commissures. This semi-annular arrangement is more evident when the female has borne children, and when the orifice of the uterus is transverse. Are the fibres of the neck of the uterus confounded with those of the vagina? Jobert thinks they are.—*Amer. Journ.*, from *Lond. and Edin. Month. Journ. Med. Sci.*, April, 1843.

